# An Innovative Patient-Centred Approach to Heart Failure Management: The Best Care Heart Failure Integrated Disease-Management Program

**DOI:** 10.1016/j.cjco.2024.03.015

**Published:** 2024-04-10

**Authors:** Christopher Licskai, Anna Hussey, Madonna Ferrone, Cathy Faulds, Melissa Fisk, Shanil Narayan, Tim O’Callahan, Andrew Scarffe, Shannon Sibbald, Dhssraj Singh, Teresa To, Jari Tuomi, Robert McKelvie

**Affiliations:** a Schulich School of Medicine and Dentistry, Western University, London, Ontario, Canada; b Asthma Research Group Windsor-Essex County Inc., Windsor, Ontario, Canada; c London Health Sciences Centre, London, Ontario, Canada; d Hotel-Dieu Grace Healthcare, Windsor, Ontario, Canada; e Huron Perth Health Care Alliance, Stratford, Ontario, Canada; f Huron Perth & Area Ontario Health Team, Stratford, Ontario, Canada; g Telfer School of Management, University of Ottawa, Ottawa, Ontario, Canada; h Faculty of Health Sciences, Western University, London, Ontario, Canada; i Windsor Heart Institute, Windsor, Ontario, Canada; j The Hospital for Sick Children, Toronto, Ontario, Canada; k ICES, Toronto, Ontario, Canada; l Dalla Lana School of Public Health, University of Toronto, Toronto, Ontario, Canada; m North Bay Regional Health Centre, North Bay, Ontario, Canada; n St. Joseph’s Health Care, London, Ontario, Canada

## Abstract

**Background:**

The management of heart failure (HF) is challenging because of the complexities in recommended therapies. Integrated disease management (IDM) is an effective model, promoting guideline-directed care, but the impact of IDM in the community setting requires further evaluation.

**Methods:**

A retrospective evaluation of community-based IDM. Patient characteristics were described, and outcomes using a pre- and post-intervention design were HF-related health-service use, quality of life, and concordance with guideline-directed medical therapy (GDMT).

**Results:**

715 patients were treated in the program (2016 to 2023), 219 in a community specialist–care clinic, and 496 in 25 primary-care clinics. The overall cohort was predominantly male (60%), with a mean age of 73.5 years (± 10.7), and 60% with HF with reduced ejection fraction. In patients with ≥ 6 months of follow-up (n = 267), pre vs post annualized rates of HF-related acute health-service use decreased from 36.3 to 8.5 hospitalizations per 100 patients per year, *P* < 0.0001, from 31.8 to 13.1 emergency department visits per 100 patients per year, *P* < 0.0001, and from 152.8 to 110.0 urgent physician visits per 100 patients per year, *P* = 0.0001. The level of concordance with GDMT improved; the number of patients receiving triple therapy and quadruple therapy increased by 10.1% (95% confidence interval [CI], 2.4%,17.8%) and 19.6% (95% CI, 12.0%, 27.3%), respectively. Within these groups, optimal dosing was achieved in 42.5% (95% CI, 32.0%, 53.6%) and 35.0% (95% CI, 23.1%, 48.4%), respectively. In patients with at least one follow-up visit (n = 286), > 50% experienced a clinically relevant improvement in their quality of life.

**Conclusions:**

A community-based IDM program for HF, may reduce HF-related acute health-service use, improve quality of life and level of concordance with GDMT. These encouraging preliminary outcomes from a real-world program evaluation require confirmation in a randomized controlled trial.

## Résumé

**Contexte:**

La prise en charge de l’insuffisance cardiaque (IC) représente un défi en raison de la complexité des traitements recommandés. La prise en charge intégrée des maladies est un modèle efficace qui favorise les soins reposant sur les lignes directrices, mais la portée de ce modèle en milieu extra-hospitalier mérite une évaluation plus approfondie.

**Méthodologie:**

Évaluation rétrospective de la prise en charge intégrée des maladies à l’échelle communautaire. Les caractéristiques des patients ont été établies. Les résultats mesurés avant et après l’intervention ont été l’utilisation des services de santé liés à l’IC, la qualité de vie et le degré de concordance avec le traitement médical recommandé par les lignes directrices.

**Résultats:**

Au total, 715 patients ont été traités dans le cadre du programme (de 2016 à 2023) : 219 dans une clinique communautaire de soins spécialisés et 496 dans 25 cliniques de soins primaires. Dans l’ensemble, la majorité des patients étaient de sexe masculin (60 %). L’âge moyen était de 73,5 ans (± 10,7 ans). Soixante pour cent des patients présentaient une IC avec fraction d’éjection réduite. Chez les patients ayant fait l’objet d’un suivi pendant ≥ 6 mois (n = 267), les taux annualisés d’utilisation des services de santé aigus liés à l’IC avant et après l’intervention sont passés de 36,3 à 8,5 hospitalisations pour 100 patients par année (*p* < 0,0001), de 31,8 à 13,1 visites aux urgences pour 100 patients par année (*p* < 0,0001) et de 152,8 à 110,0 consultations avec un urgentologue pour 100 patients par année (*p* = 0,0001). On a observé une augmentation du degré de concordance avec le traitement médical recommandé par les lignes directrices. Le nombre de patients recevant une trithérapie et une quadrithérapie a également augmenté de 10,1 % (intervalle de confiance [IC] à 95 % : 2,4 % à 17,8 %) et de 19,6 % (IC à 95 % : 12,0 % à 27,3 %), respectivement. Dans ces groupes, la dose optimale a été atteinte chez 42,5 % (IC à 95 % : 32,0 % à 53,6 %) et 35,0 % (IC à 95 % : 23,1 % à 48,4 %) des patients, respectivement. Plus de 50 % des patients ayant effectué au moins une visite de suivi (n = 286) ont obtenu une amélioration cliniquement pertinente de leur qualité de vie.

**Conclusions:**

Un programme communautaire de prise en charge intégrée de l’IC peut réduire l’utilisation des services de santé liés à l’IC, améliorer la qualité de vie et augmenter le degré de concordance avec le traitement médical recommandé par les lignes directrices. Bien qu’ils soient encourageants, ces résultats préliminaires issus de l’évaluation d’un programme en contexte réel doivent être confirmés par la réalisation d’une étude à répartition aléatoire et contrôlée.

Heart failure (HF) is a chronic progressive syndrome; it is the second most common reason for admission to the hospital for Canadians aged > 65 years, and it is the leading cause of cardiovascular morbidity and mortality.^1^, ^2^, ^3^ In Canada, the number of people aged > 40 years who are living with HF has increased, from 467,940 in 2000 to 798,675 in 2020.^4^ The personal and health-system ramifications of HF in Canada are substantial. Despite improvement in therapies over the past decade, high levels of health-system utilization and expenditure remain constant.^3^

HF management is complex and resource-intensive. Meta-analyses have demonstrated that multidisciplinary integrated disease-management (IDM) programs characterized by self-management strategies, education, guideline-directed medical therapy (GDMT) optimization, and case management reduce all-cause and HF-related mortality and hospitalizations rates.^5^, ^6^, ^7^, ^8^, ^9^ Currently, only very limited access to HF-related IDM is available in Canada. Thus, implementing integrated clinical pathways for people with HF has been identified as a priority within health systems in Canada.^10^ Most patients with HF are managed in the community by their primary-care provider, but of the studies identified by systematic reviews, only 5 were conducted in Canada, and none was conducted in a Canadian primary-care HF cohort.^2^^,^^5^, ^6^, ^7^, ^8^, ^9^ Despite an emerging consensus that enhanced involvement of primary-care services in HF management is key to managing this growing patient population, evidence to fully support this strategy is lacking.^9^^,^^11^^,^^12^

Pharmacologic therapies are a pivotal component of HF management. Four pillars for GDMT have been identified for patients with HF with reduced ejection fraction (HFrEF), as follows: (i) angiotensin-converting enzyme inhibitor (ACEi)/ angiotensin II receptor blocker (ARB)/ angiotensin receptor–neprilysin inhibitor (ARNI); (ii) beta-blockers; (iii) mineralocorticoid receptor antagonists (MRAs); and (iv) sodium glucose cotransporter-2 inhibitors (SGLT2i).^13^, ^14^, ^15^, ^16^, ^17^ GDMT is based on results from major landmark clinical trials, and the greatest clinical benefits (reduced mortality and reduced hospital admissions) are seen when all 4 pillar drugs are used together and are titrated to an optimal dose.^11^^,^^13^^,^^15^^,^^17^ However, despite the strong evidence base, only a minority of individuals with HFrEF are receiving all 4 drugs concurrently and at optimal dosing.^14^^,^^18^ Approximately half of patients in the community who have the signs and symptoms of HF have HF with a preserved ejection fraction (HFpEF).^19^ Therapeutic strategies for this population have focused on the treatment of comorbidities and symptom management.^15^ However, similar to results found for patients with HFrEF, more recent studies have demonstrated that adherence to GDMT, including a SGLT2i, reduces the risk of cardiovascular death or hospitalization in patients with HFpEF.^20^^,^^21^ In selected patients with HFpEF, the use of an MRA and an ARB may reduce the incidence of clinical events.^15^

Access to IDM is limited, and evidence that community-based IDM programs are effective is lacking. The Best Care HF program is an IDM program embedded within primary-care clinics and a community specialist-care clinic in Ontario, Canada. The purpose of this study was to evaluate the Best Care HF program, by identifying the population it serves and investigating changes in HF-related health-service use (HSU), health-related quality of life (QoL), and pharmacologic management.

## Methods

### Study design and objectives

Using a pre–post study design, we conducted a retrospective evaluation of the Best Care program using patients managed in primary care and a community-based specialist-care clinic in Ontario, Canada, between May 31, 2016 and February 28, 2023. The objectives were as follows: (i) to characterize the community-based population with HF enrolled in the program; (ii) to investigate change in pre-program and post-program HF-related hospital admissions, emergency department (ED) visits, and urgent family physician visits; (iii) to assess change in QoL; and (iv) to examine change in GDMT in patients with HFrEF. The Veritas Independent Review Board approved the study (refernce number: 2023-3218-14132-2).

### Inclusion criteria

To describe the largest possible cohort, all patients enrolled in the Best Care HF program (May 31, 2016 to February 28, 2023) were included. To capture acute HSU, patients in the Best Care HF program cohort with a minimum of 6 months of follow-up postintervention were included in the analysis. A minimum of 6 months was chosen to reduce the bias associated with rate estimates for patients with only a few months of follow-up data. Patients in the cohort with a minimum of 1 follow-up appointment postintervention were included in the analysis for QoL. Patients from the cohort with HFrEF and a minimum of 6 months of follow-up postintervention were included to assess the change in GDMT. Reasons for exclusion were investigated and reported.

### Best Care HF program

The goal of the Best Care HF program is to deliver all elements of evidence-based best practices. The most common configuration of the program has been one that is embedded within a primary-care clinic, either within a group practice or alongside a solo practitioner. However, since 2020, the Best Care HF program also has provided support to a community-based specialist-run cardiology clinic, and data from both the primary-care clinic and the specialist-care clinic have been included in this study. Details of the Best Care HF program have been described previously.^22^ In brief, the program utilizes a team-care triad consisting of the patient, a cardiac educator–case manager (CEC), and a healthcare practitioner. The healthcare practitioner in this study was the primary-care practitioner in the primary-care clinic, or a cardiologist in the specialist-care clinic.

Patients were identified by practice audit, using electronic health record searches, or were referred to the program by their healthcare practitioner. Patients included were those with a clinical diagnosis of HF, differentiated as either HFpEF or HFrEF by an echocardiogram or another clinically accepted technique to measure left ventricular ejection fraction.

Patients referred to the program were evaluated comprehensively, in person, by the CEC, during an initial visit lasting 60-90 minutes, on site at their primary-care or specialist-care clinic. Follow-up visits were arranged depending on patients’ needs; the average number was 3-4 appointments per year (30-45 minutes). The CEC assessment included diagnostic confirmation, case management, medication management (review, titration, and optimization), skills training, and self-management education, including a diuretic action-plan. The CEC then consulted with the patient’s healthcare practitioner in real time, to finalize, approve, and implement needed pharmacologic and nonpharmacologic interventions, and determine if specialty referral was required. The Best Care HF program is not a time-limited intervention but rather a continuous chronic disease–care program. The Best Care program intervention is standardized by means of a custom-designed electronic health record that has embedded program standards, is integrated into clinical workflow to guide every patient encounter, and collects and stores patient data.

### Data collection

Baseline demographic and clinical characteristics were collected at the initial visit for all patients on the program. The data collected were age, sex, racial group, body mass index, smoking status, age-adjusted Charlson Comorbidity Index (CCI), New York Heart Association (NYHA) functional classification, prior-year acute HF-related HSU (hospital admissions, ED visits, and urgent family-physician visits), comorbidities, and current HF medications.^23^, ^24^, ^25^, ^26^, ^27^, ^28^ In the earlier years of the Best Care HF program, the Minnesota Living with HF Questionnaire (MLHFQ) was used to measure QoL; a change was made to using the Kansas City Cardiomyopathy Questionnaire (KCCQ) in 2018. The KCCQ is a validated 23-item disease-specific questionnaire, scored from 0 to 100, with higher scores indicating better health status or QoL.^26^ The MLHFQ is a validated 21-item disease-specific questionnaire, scored from 0 to 105, with higher scores indicating poorer health status or QoL.^28^ For both tools, a change of 5 points is considered the minimum clinically important difference (MCID).^26^, ^27^, ^28^ The NYHA classification and a QoL measurement (using either the MLHFQ or the KCCQ) were collected at most patient encounters.

### Outcomes

We predefined clinically relevant outcomes, including acute HF-related (HSU), disease-specific QoL (KCCQ or MLHFQ), and concordance of pharmacologic management with GDMT. Acute HF-related HSU was self-reported, validated by medical record auditing, and it included hospital admissions, ED visits, and urgent family-physician visits. Urgent family-physician visits refer to nonroutine appointments required for HF symptoms. Hospital admissions and ED visits were mutually exclusive (if an ED visit led to a hospital admission, it was recorded as a hospital admission only). The mean QoL scores over the follow-up interval were compared to the baseline values. GDMT for patients with HFrEF compared the medications at the initial visit to the medications at the most recent appointment. Patient status was categorized according to whether target doses were achieved, as follows: optimized to guidelines, optimized to tolerance, actively titrating, and not optimized.

### Statistical analyses

Baseline characteristics were presented as continuous variables (mean ± standard deviation) or as categorical variables (frequency and percentage) for the overall study population, and they were classified by primary-care IDM and specialist-care IDM. Pre–post differences in outcomes were investigated for normalcy in distribution and compared using a paired Student *t* test, a Wilcoxon signed rank test, or a McNemar test, as appropriate. A *P* value < 0.05 was considered statistically significant; a Holm correction was applied to account for multiple testing, and 95% confidence intervals (CIs) are reported.^29^ Hospital admission, ED visit, and urgent family-physician visit rates (events per 100 patients per year) were calculated using the number of events in the year prior and were compared to the annualized number of events over the follow-up period.

Change in QoL measured by the KCCQ or the MLHFQ was determined as the baseline score minus the mean of all documented follow-up scores (within-patient measurements included only one QoL tool). Patients were grouped by level of change (improved, stable, or worsening QoL), using a 5-point MCID for both tools. Stratification by baseline QoL category quartiles (good, moderate, poor, and very poor) was performed to further explore change in QoL. GDMT at baseline was compared to treatment at the last follow-up visit. Pharmacologic optimization was investigated by comparing target dosing of HF medications at the initial visit with target dosing at the most recent visit.

### Sensitivity analyses

Asymmetric recruitment, and the COVID-19 pandemic, combined with the retrospective real-world design of the evaluation meant that 35%-40% of the total cohort were eligible for the outcome analysis. To identify any selection bias that may have been present, we performed 2 sensitivity analyses. In the first analysis, baseline characteristics of patients excluded from the HSU outcome analyses with < 6 months of follow-up were compared to characteristics for those included in the analyses. In the second sensitivity analysis, baseline characteristics of patients excluded due to incomplete QoL data were compared to those of patients included in the QoL outcome analysis. Patients recently enrolled in the program who simply had not had enough time in the study period to meet the inclusion criteria were not included in these sensitivity analyses, as there was no reason to assume the presence of any systematic differences from the cohort included in the outcome analyses. Additional post hoc sensitivity analyses were performed to investigate whether the setting (primary-care or specialist-care) or HF type (HFrEF vs HFpEF) were dominating the observed results; stratified analyses for acute HSU and QoL were repeated, first by setting, and second by HF type.

Statistical analyses were performed using Stata/MP 17.0 (StataCorp, College Station, TX).

## Results

### Characteristics of the study population

**Figure 1:**
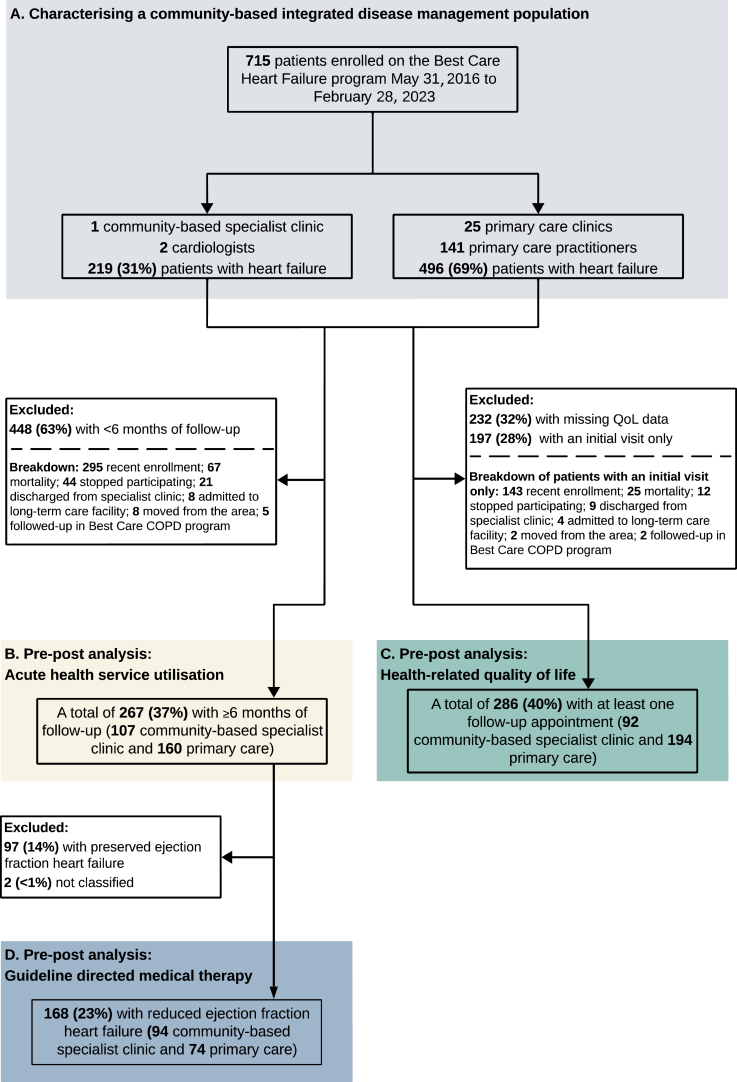
Flow diagram of the study showing the analyses groups. COPD, chronic obstructive pulmonary disorder; QoL, quality of life.

From May 2016 to February 2023, 715 individuals were enrolled in the Best Care HF program (Fig. 1A). Of these, 219 (30.6%) were enrolled in the community specialist-care clinic involving 2 cardiologists, and 496 (69.4%) were enrolled in 25 primary-care clinics involving 141 primary-care practitioners. The follow-up period, in patients with more than one appointment, ranged from 3 months to > 6 years (median, 7.5 months).

The overall HF population (N = 715) included predominantly male patients, 59.6%, with a mean age of 73.5 years (± 10.7), a body mass index of 31.5 kg/m^2^ (± 7.7), and a smoking prevalence of 9.0% (Table 1). The mean age-adjusted CCI was 5.4 (± 1.9), and 81.1% had more than 2 comorbidities. The number of patients with HFrEF was greater (60.0%) than the number with HFpEF (38.3%), and > 80% were categorized as NYHA class II or III. A total of 263 hospital admissions (36.8 per 100 patients per year), 214 ED visits (29.9 per 100 patients per year), and 924 urgent family-physician visits (129.2 per 100 patients per year) related to HF were completed in the year prior to the initial visit.

**Table 1:** Baseline demographic and clinical characteristics of all patients at time of entry into integrated disease management for HF

Baseline demographic and clinical characteristics	Complete cohort n = 715	Specialist-care clinic n = 219	Primary-care clinic n = 496
Sex			
Male	426 (59.6)	143 (65.3)	283 (57.1)
Female	289 (40.4)	76 (34.7)	213 (42.9)
Age, y, mean (SD)	73.5 (10.7)	71.6 (11.9)	74.3 (10.0)
Body mass index, kg/m^2^, mean (SD)	31.5 (7.7)	30.9 (7.5)	31.8 (7.8)
Racial group, Caucasian	695 (97.2)	209 (95.4)	486 (97.9)
Smoking status, current smoker	64 (9.0)	19 (8.7)	45 (9.1)
Quality of life			
KCCQ (score 0–100),∗ mean (SD)	66.6 (24.6)	63.0 (25.8)	70.0 (22.9)
MLHFQ (score 0–105),† mean (SD)	29.1 (20.2)	NR¶	28.8 (19.9)
Missing	84 (11.7)	15 (6.8)	72 (14.6)
Comorbidities			
0	10 (1.4)	2 (0.9)	8 (1.6)
1–2	125 (17.5)	28 (12.8)	97 (19.6)
> 2	580 (81.1)	189 (86.3)	391 (78.8)
Charlson Comorbidity Index‡			
Mean (SD)	5.4 (1.9)	5.4 (2.1)	5.3 (1.7)
≥ 5	328 (69.5)	151 (69.6)	177 (69.4)
Seen by specialist			
Cardiologist	454 (63.5)	219 (100)	235 (47.4)
Internal medicine	124 (17.3)	24 (11.0)	100 (20.2)
None	185 (25.9)	-	185 (37.2)
HFrEF, LVEF ≤ 45%	429 (60.0)	191 (87.2)	238 (48.0)
HFpEF, LVEF > 45%	274 (38.3)	28 (12.8)	246 (49.6)
Missing	12 (2.0)	-	12 (2.4)
Echocardiogram year prior	616 (86.2%)	211 (96.4)	405 (81.7)
NYHA class			
I	119 (16.6)	47 (21.5)	72 (14.5)
II	356 (49.8)	98 (44.8)	258 (52.0)
III	221 (30.9)	70 (32.0)	151 (30.4)
IV	19 (2.7)	4 (1.8)	15 (3.0)
HF-related health-service use (year prior)			
Hospital admissions			
Number of events	263	88	175
Number of individuals	202 (28.3)	65 (29.7)	137 (27.6)
Rate of events per 100 patients per y	36.8	40.2	35.2
Emergency department visits (not leading to admission)			
Number of events	214	63	151
Number of individuals	154 (21.5)	42 (19.2)	112 (22.6)
Rate of events per 100 patients per y	29.9	28.8	30.4
Urgent family-physician visits			
Number of events	924	219	705
Number of individuals	323 (45.2)	128 (58.4)	195 (39.3)
Rate of events per 100 patients per y	129.2	100.0	142.1
Medications			
ARNI (HFrEF only),§	186 (43.4)	118 (61.8)	68 (28.6)
ACEi/ARB	293 (41.0)	60 (27.4)	233 (47.0)
Beta-blocker	540 (75.5)	203 (92.7)	337 (67.9)
MRA	254 (35.5)	118 (53.9)	136 (27.4)
SGLT2i	168 (23.5)	88 (40.2)	80 (16.1)
Diuretic	496 (69.4)	149 (68.0)	347 (70.0)

Values are n (%), unless otherwise indicated.

ACEi, angiotensin-converting enzyme inhibitor; ARB, angiotensin receptor blocker; ARNI, angiotensin receptor/neprilysin inhibitor; HF, heart failure; HFpHF, HF with preserved ejection fraction; HFrEF, HF with reduced ejection fraction; KCCQ, Kansas City Cardiomyopathy Questionnaire; LVEF, left ventricular ejection fraction; MLHFQ, Minnesota Living with Heart Failure Questionnaire; MRA, mineralocorticoid receptor antagonist; NR, not reported; NYHA, New York Heart Association;.SD standard deviation; SGLT2i, sodium-glucose cotransporter-2 inhibitor.

∗ KCCQ-23, scored 0–100, with 100 representing best quality of life. N = 423; n = 202 (specialist-care clinic); n = 221 (primary-care clinic).

† MLHFQ, scored 0–105 with 105 representing the worst quality of life. N = 208, n = 206 (primary-care clinic).

‡ Charlson Co-morbidity Index self-reported since September 2020; N = 472; n = 217 (specialist-care clinic); n = 255 (primary-care clinic). (Age-adjusted index reported.)

§ Only HFrEF, N = 429; n = 191 (specialist-care clinic); n = 238 (primary-care clinic).

¶ NR, as sample size too small.

A comparison of baseline patient characteristics in the specialist-care clinic to those in the primary-care clinics showed that patients were on average younger (71.6 years [ ± 11.9] vs 74.3 years [ ± 10.0]), had a numerically worse QoL score (KCCQ score, 63.0 (± 25.8) vs 70.0 (± 22.9), and that a higher proportion of patients had HFrEF (87.2% vs 48.0%). The specialist-care clinic cohort had a similar proportion of hospitalizations (29.7% vs 27.6%) and ED visits (19.2% vs 22.6%), but it had a higher number of urgent family-physician visits in the year prior (58.4% vs 39.3%). The primary-care and specialist-care clinics were managing patients with an equal level of comorbidities (mean CCI: 5.4 [ ± 2.1] vs 5.3 [ ± 1.7]). Overall, the level of concordance with GDMT for HFrEF was higher in the specialist-care clinic group (beta-blocker [92.7% vs 67.9%], MRA [53.9% vs 27.4%], and SGLT2i [40.2% vs 16.1%]). The level of ARNI use was higher in the HFrEF specialist-care clinic patients (61.8% vs 28.6%), and by corollary, the level of ACEi/ARB use (27.4% vs 47.0%) was lower (Table 1).

### Acute HF-related HSU

**Figure 2:**
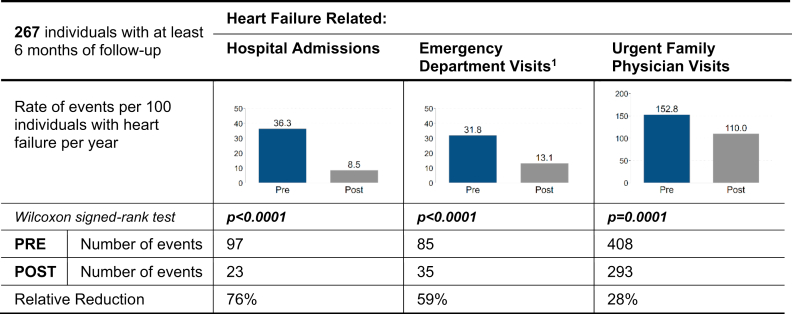
Acute health-service use for heart failure, comparing the year prior to the Best Care program with the annualized year post–Best Care program. PRE indicates the year prior to commencing Best Care integrated disease management. POST indicates the year after enrollment in Best Care integrated disease management. Number of events is calculated from the rate ([rate × number of patients]/100). Rate is annualized ([events/months of follow-up] × 12 × 100). Significance level is 0.05; *P* values adjusted for multiple testing using the Holm correction. Bold indicates significance. ∗Visits to the emergency department that did not result in a hospital admission.

A total of 267 individuals (37.3%) met the inclusion criteria of at least 6 months of follow-up, for inclusion in these analyses (Fig. 1B). In the year prior to enrolling in the Best Care program, patients had 97 hospital admissions (36.3 per 100 patients per year), 85 ED visits (31.8 per 100 patients per year), and 408 urgent family-physician visits (152.8 per 100 patients per year; Fig. 2). Annualized event rates post–Best Care enrollment were significantly lower, at 23 hospital admissions (8.5 per 100 patients per year, *P* < 0.0001), 35 ED visits (13.1 per 100 patients per year, *P* < 0.0001), and 293 urgent family-physician visits (110.0 per 100 patients per year, *P* = 0.0001). Stratified analyses confirmed consistent findings within all subgroups, including specialist-care and primary-care, HFrEF, and HFpEF (Supplemental Figs. S1-S8).

### Health-related QoL

**Figure 3:**
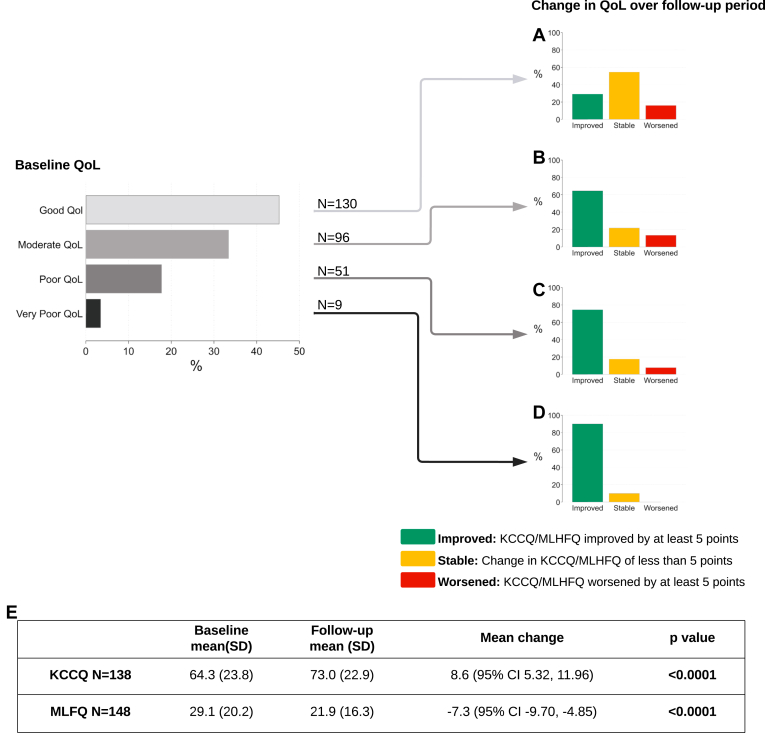
Change in health-related quality of life (QoL), stratified by baseline score, in the 286 individuals with documented QoL scores at initial visit and at least one follow-up visit. Baseline QoL score categorized by quartile of questionnaire scoring range: Kansas City Cardiomyopathy Questionnaire (KCCQ; good, 75-100; moderate, 50-74; poor, 25-49; very poor, < 25); and Minnesota Living with Heart Failure Questionnaire (MLHFQ; good, < 26; moderate, 26-52; poor, 53-79; very poor, 80-105). Change in QoL is the proportion of individuals who experienced a clinically relevant change over the follow-up period. Results are stratified by QoL at baseline: (**A**) good; (**B**) moderate; (**C**) poor; (**D**) very poor. A change of ≥ 5 points was considered clinically relevant. A mean of all follow-up values was taken and subtracted from the baseline score. (**E**) Data table gives the mean change in QoL from baseline, and a paired difference test of repeated measures (Wilcoxon signed-rank test). Significance level is 0.05; *P* values are adjusted for multiple testing using the Holm correction. Bold indicates significance. CI, confidence interval; SD, standard deviation.

A total of 286 individuals (40.0%) met the inclusion criteria, having a QoL score at the initial visit, and at least one follow-up score (Fig. 1C). The mean change from baseline for KCCQ score and MLHFQ score showed improvement surpassing the MCID of 5 points, with the KCCQ score change at 8.6 points (CI, 5.32,11.96) and the MLHFQ score change at –7.3 points (CI, -9.70,–4.85). Baseline categorization of QoL scores demonstrated that 45.5% of individuals had a good QoL score, 33.5% had a moderate baseline QoL score, and nearly 20% had a poor or very poor QoL score. The change in QoL was greatest for patients with poor or very poor baseline QoL scores, with a clinically relevant improvement in 74.5% and 88.9% of patients, respectively (Fig. 3). Stratified analyses confirmed consistent findings within all subgroups, including specialist-care and primary-care, HFrEF, and HFpEF (Supplemental Figs. S1-S8).

### Pharmacologic management by pillar category

Pharmacologic management outcomes were analyzed for 168 patients (23.5%) who met the inclusion criteria of having HFrEF and at least 6 months of follow-up (Fig. 1D). The proportions of patients increased as follows: those on an ARNI by 15.4% (CI, 8.3%, 22.7%); those on an MRA by 11.3% (CI, 3.8%,18.8%); those on an SGLT2i by 19.0% (CI, 11.7%, 26.4%), those on triple therapy by 10.1% (CI, 2.4%, 17.8%), and those on quadruple therapy by 19.6% (CI, 12.0%, 27.3%; Table 2). The proportion of patients on an ACEi and/or ARB decreased by –15.4% (CI, -22.9%, –8.1%), indicating a within-class switch from use of an ACEi and/or ARB to use of an ARNI. No significant change occurred in the proportion of patients on beta-blockers.

**Table 2:** Pharmacologic management of individuals with heart failure with reduced ejection fraction (HFrEF) at baseline vs their most-recent follow-up

	HFrEF			
	Initial visit N = 168	Most recent visit N = 168	% difference (95% CI)∗	***P***
ARNI	65 (38.7)	91 (53.9)	**15.4 (8.3, 22.7)**	**< 0.0001**
ACEi/ARB	66 (39.3)	40 (23.8)	**–15.4 (–22.9, –8.1)**	**< 0.0001**
ACEi/ARB/ARNI	131 (78.0)	131 (78.0)	0	
Beta-blocker	146 (87.0)	149 (88.7)	1.8 (–3.0, 6.6)	0.5811
MRA	82 (48.8)	101 (60.1)	**11.3 (3.8, 18.8)**	**0.0026**
SGLT2i	42 (25.0)	74 (44.1)	**19.0 (11.7, 26.4)**	**< 0.0001**
Triple therapy†	70 (41.7)	87 (51.8)	**10.1 (2.4, 17.8)**	0.0095
Quadruple Therapy‡	27 (16.1)	60 (35.7)	**19.6 (12.0, 27.3)**	**< 0.0001**

**Figure 4:**
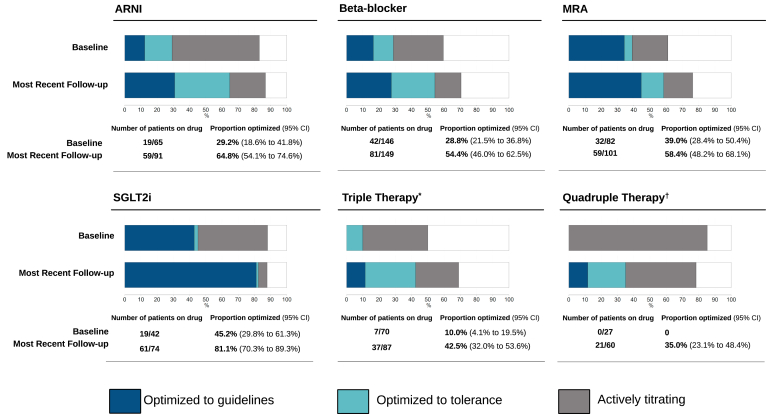
Optimization of the pharmacologic management of individuals with reduced ejection fraction heart failure at baseline vs at their most-recent follow-up. The numerator is the number of patients on the drug for whom the dosage has been optimized to target or tolerance; the denominator is the number of people on the drug. ARNI, angiotensin receptor/neprilysin inhibitor; CI confidence interval; MRA mineralocorticoid receptor antagonist; SGLT2i, sodium–glucose cotransporter-2 inhibitor. ∗ ARNI/ACEi/ARB, beta-blocker, and MRA. ^†^ ARNI/ACEi/ARB, beta-blocker, MRA, and SGLT2i.

This table shows the number and proportion of patients, n (%), on key guideline-directed pharmacologic therapies for HFrEF, the data in Figure 4 builds from these proportions. For example, 16% of patients are on quadruple therapy at initial visit (Table 2), and of those, 0% are at target dose (Fig. 4). Data are missing for 2 individuals. Only individuals with at least 6 months of follow-up were included. Significance level is 0.05; *P* values are adjusted for multiple testing using the Holm correction. Bold indicates significance.

ACEi, angiotensin-converting enzyme inhibitor; ARB, angiotensin receptor blocker; ARNI, angiotensin receptor/neprilysin inhibitor; CI, confidence interval; MRA, mineralocorticoid receptor antagonist; SGLT2i, sodium–glucose cotransporter-2 inhibitor.

∗ McNemar’s χ^2^ test used to compare pre–post differences for patients diagnosed with HFrEF at initial visit, n = 168. *P* value refers to the exact McNemar significance probability.

† Triple therapy included to reflect changing guidelines over the follow-up period. Patients on an ARNI, beta-blocker, and MRA (ACEi and/or ARB instead of an ARNI also considered triple therapy).

‡ Patients on an ARNI, beta-blocker, MRA, and SGLT2i (ACEi and/or ARB instead of an ARNI also considered quadruple therapy).

### Pharmacologic optimization within each pillar

Pharmacologic optimization increased for all 4 pillar HF drugs used for individuals with HFrEF (Fig. 4). The percentage of patients taking the drug at the optimal dosage (guideline target or limit of dose tolerance) increased as follows from baseline to the most recent visit: for ARNIs, from 29.2% (CI, 18.6%, 41.8%) to 64.8% (CI, 54.1%, 74.6%); for beta-blockers, from 28.8% (CI, 21.5%, 36.8%) to 54.4% (CI, 46.0%, 62.5%); for MRAs, from 39.0% (CI, 28.4%, 50.4%) to 58.4% (CI, 48.2%, 68.1%); for SGLT2is, from 45.2% (CI, 29.8%, 61.3%) to 81.1% (CI, 70.3%, 89.3%); for triple therapy, from 10.0% (CI, 4.1%, 19.5%) to 42.5% (CI, 32.0%, 53.6%), and for quadruple therapy, from 0% to 35.0% (CI, 23.1%, 48.4%).

### Sensitivity analyses

#### Less than 6 months of follow-up

A total of 153 patients (21.4%) were excluded from the acute HSU outcome analyses, owing to insufficient follow-up that was not related to recent program enrollment (67 died; 44 stopped participating in the program; 21 were discharged from the specialist-care clinic; 8 left their primary-care practice; 8 were admitted to a long-term care facility; and 5 received follow-up in the Best Care chronic obstructive pulmonary disease program; Fig. 1). This excluded group had a higher predominance of female patients (51.6% vs 38.6%), a higher proportion of patients with HFpEF (50.3% vs 36.6%), and higher rates (events per 100 patients per year) of hospital admissions and ED visits (46.4 vs 36.3 and 40.5 vs 31.8, respectively), compared to the numbers for the 267 patients included in the outcome analysis (Supplemental Table S1).

#### Missing QoL data

A total of 232 patients (32.4%) had missing QoL scores at baseline and/or over their follow-up period. No notable differences were observed between individuals with missing QoL scores and the 287 patients included in the outcome analysis (Supplemental Table S2). The 197 patients who did not have missing data, but had only an initial visit were not included in the sensitivity analyses (see Fig. 1 for a full breakdown of exclusions).

## Discussion

We identified and characterized more than 700 patients with HF in a Canadian community-practice setting, including nearly 500 from primary-care practices. The primary-care population was an elderly comorbid cohort, with a moderately reduced QoL, exercise-limiting dyspnea, almost equal proportions of HFrEF and HFpEF patients, and high rates of hospitalization in the prior year. Although the community specialist-care population had a poorer baseline QoL score, and a higher proportion of HFrEF patients, the populations were remarkably similar. Both community clinical settings were managing complex comorbid patient populations with a high mean CCI that was similar in both groups. Annualized rates of hospitalizations, ED visits, and unscheduled urgent family-physician visits for HF were reduced significantly following Best Care HF program implementation. Similarly, marked improvements occurred in QoL. In patients with HFrEF, the level of concordance with GDMT increased. Stratified subset analyses confirmed consistent findings in all of the main outcomes within all subgroups, including specialist-care and primary-care, HFrEF, and HFpEF.

The spoke–hub–node model describes a system of HF care with vertical integration from primary care to quaternary care with provider roles that are defined based on services provided according to their patients’ medical complexity.^12^ In the spoke, patients of lower complexity can be effectively managed without involvement of a multidisciplinary team. The node manages the most-complex patients with a multidisciplinary HF team. The hub manages patients of moderate complexity, such as those included in this study. The findings of this study suggest that the Best Care program can support a primary-care or a community specialist-care clinic to effectively function as a hub to manage moderately complex patients with HF.^12^

IDM is an accepted standard of care in the management of HF. In a recent systematic review, Takeda and colleagues evaluated IDM that was implemented after a patient hospitalization.^7^ They included 47 randomized controlled trials with 10,869 participants and found moderate-quality evidence that case management and multidisciplinary interventions reduce HF readmissions (risk ratio, 0.64, 95% CI, 0.53-0.78, or 36% risk reduction; and risk ratio, 0.68, 95% CI, 0.50- 0.92, or 32% risk reduction, respectively).These interventions included key elements that are also central to the Best Care program; they used case managers to actively manage care and featured coordinated healthcare interventions, such as self-management strategies.^7^ Acknowledging the different methodologies, in this study, we demonstrated a 76% relative risk reduction in hospitalization events. A notable point is that most IDM programs evaluated to date are reactive, targeting patients discharged from the hospital. This study adds to the literature by examining an “upstream” approach, whereby patients with HF were proactively identified and managed in an outpatient community setting. Also similar to our findings, and using pre–post data, Liljeroos and Strömberg found that nurse-led primary-care HF clinics in Sweden reduced the incidence of ED visits and the need for inpatient care, by 24% and 27%, respectively.^30^ Likewise, in a randomized controlled trial, Agvall and colleagues found that a HF disease-management program involving family physicians and HF nurses in primary care significantly reduced the incidence of ED visits and hospital admissions, as compared to the incidence in the usual-care group.^31^

GDMT is recommended uniformly in HF guidelines, but despite this universal recommendation, patients with HF remain undertreated.^14^^,^^32^^,^^33^ In this study, we use GDMT as an indicator of the therapeutic care gap for patients with HFrEF, and as a marker of change in guideline concordance postintervention. Guideline-directed triple therapy for patients with HFrEF was recommended in Canada in 2017, and quadruple therapy was recommended in 2021.^1^^,^^7^ Less than 45% of our HF patients were receiving triple GDMT, and of those patients, 10% received dosages that were optimized based on guidelines or tolerance at baseline. A minority (16.1%) were on quadruple GDMT therapy at baseline, and none had dosages optimized to target or to tolerance. The care gap identified in our population has been observed in other studies.^32^, ^33^, ^34^ This finding further emphasizes the importance of identifying management strategies that can effectively optimize GDMT.

The Best Care program CECs support medication up-titration as a program standard, adopting coordinated titration strategies encouraging well-timed optimization.^35^ This study reports marked improvements in optimization of GDMT to target or tolerance after the Best Care intervention. Related to the real-world retrospective study design, a high proportion of patients still were having their medications actively titrated at the time of data analysis. In a Canadian hospital-based multidisciplinary HF clinic study, the proportions of patients receiving HFrEF pharmacologic therapies after 6 months of enrollment were as follows: 52%, for ARNIs; 97%, for beta-blockers; and 85%, for MRAs.^36^ In our study cohort, the proportions were 54%, 89%, and 60%, respectively. In the same study, population medication optimization (to target or tolerance) was reported within these drug groups, at 63% for ARNIs, 68% for beta-blockers, and 59% for MRAs; comparatively, in our study, a respective 65%, 54%, and 58% were optimized.^34^ For pharmacologic combination therapy, these authors report 77% receiving triple therapy, with 33% having therapy medically optimized; we found that 52% were on triple therapy, with medical optimization being achieved for 43%.^34^^,^^36^ The substantial improvements reported in the Best Care community program align with the magnitude of improvement in GDMT observed in a multidisciplinary hospital-based HF clinic. This finding is noteworthy in that it reinforces the important role that primary-care and community-based specialist-care clinics, with the support of the Best Care intervention, can play in narrowing the systemwide gap in achieving GDMT.

This retrospective observational study had a preintervention, postintervention design. Without a randomized comparator arm, we are unable to determine whether a causal relationship exists between the Best Care program and the reported outcomes. We cannot exclude the possibility that regression-to-the-mean bias impacted our results; however, we identified patients in a nonacute outpatient setting, to some extent mitigating this factor. We performed the pre–post analysis on patients with available data (QoL, n = 286) and those who had at least 6 months of follow-up (HSU, n = 267; pharmacologic management, n = 168). Therefore, to investigate potential selection bias, we assessed baseline characteristics of the patients with missing QoL data, and those not completing at least 6 months of follow-up, and mostly minimal differences between groups were observed. The excluded population had some features of increased severity in that mortality was the predominant reason for exclusion, and this group had a higher baseline rate of acute HSU (hospital admissions and ED visits). Thus, if included, this group may have moderated the measured impact. Further, we cannot exclude the possibility that other interventions have impacted our results, but we are not aware of other interventions available to our cohort, outside of usual care. To confirm that our results were not dominated by the outcomes of the Best Care HF program embedded in the specialist-care clinic, we stratified the analyses separating the community specialist-care and primary-care practices and found consistent pre–post improvements in both strata. This finding indicates that the practitioner types (primary-care vs specialist) had equal impacts. We included patients who were enrolled in the program during the COVID-19 pandemic, and we cannot exclude the possibility that the pandemic impacted the outcomes. Despite the identified limitations, our study provides an important empirical evaluation in favour of the Best Care HF program, evidence that is otherwise absent in relation to the Canadian healthcare system. Areas for future research include a cluster randomized controlled trial that is currently underway to establish whether a causal relationship between IDM and improved outcomes does indeed exist.^22^

## Conclusion

This study describes a pre–post evaluation of the Best Care IDM program used in community-based primary care and specialist care to manage patients with HF. In this preliminary investigation of the Best Care HF program, we observed reductions in the incidence of hospitalizations, ED visits, and urgent physician visits, with improvements in QoL and concordance with GDMT. These findings support the implementation of IDM in primary-care and specialist-care settings.
