# Supplementary material for: An Innovative Patient-Centred Approach to Heart Failure Management: The Best Care Heart Failure Integrated Disease-Management Program
Source: CJC Open. 2024 Apr 10;6(8):989–1000. doi: 10.1016/j.cjco.2024.03.015 (PMC11357758; doi:10.1016/j.cjco.2024.03.015)
Supplement: Supplemental Figures and Tables [file mmc1.docx]

**Supplemental Figures and Tables**

**Supplemental Figure S1** Acute health service use for heart failure, comparing the year prior to Best Care with annualised year post in community-based **specialist care**.

| **107** individuals with at least 6 months of follow-up | | **Heart Failure Related:** | | | | | |  |
| --- | --- | --- | --- | --- | --- | --- | --- | --- |
|  |  | **Hospital Admissions** | | **Emergency Department Visits^1^** | | **Urgent Family Physician Visits** | | |
| Rate of events per 100 individuals with heart failure per year | | 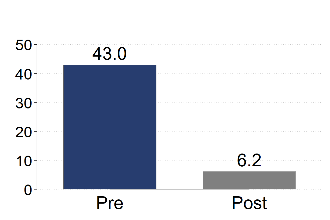 | 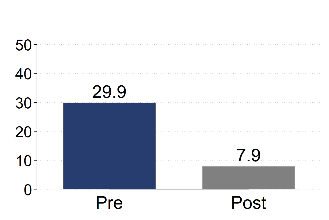 | | 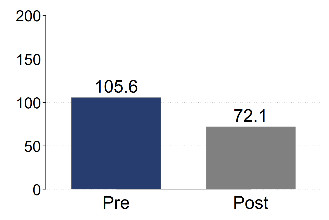 | |  |  |
| *Wilcoxon signed-rank test* | | ***p<0.0001*** | ***p=0.0025*** | | *p=0.0726* | |  |  |
| **PRE** | Number of events | 46 | 32 | | 113 | |  |  |
| **POST** | Number of events | 7 | 8 | | 77 | |  |  |
| Relative Reduction | | 85% | 75% | | 32% | |  |  |
| **Notes**:  **PRE**: year prior to commencing IDM  **POST:** year after IDM enrolment. Number of events is calculated from the rate ((rate*number of patients)/100), Rate is annualised ((events/ months of follow-up) *12*100)  **^1^**Visits to the emergency department that did not result in a hospital admission.  Significance level 0.05, p values adjusted for multiple testing using the Holm correction, bold indicates significance. | | | | | | | |  |

**Supplemental Figure S2**: Acute health service use for heart failure, comparing the year prior to Best Care with annualised year post in **primary care**.

| **160** individuals with at least 6 months of follow-up | | **Heart Failure Related:** | | | | | |  |
| --- | --- | --- | --- | --- | --- | --- | --- | --- |
|  |  | **Hospital Admissions** | | **Emergency Department Visits^1^** | | **Urgent Family Physician Visits** | | |
| Rate of events per 100 individuals with heart failure per year | | 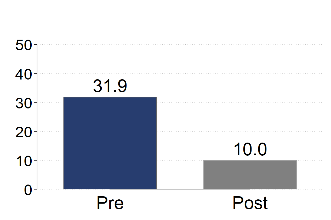 | 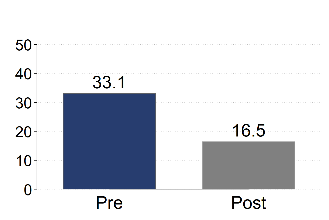 | | 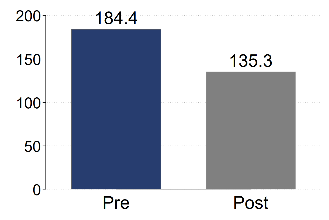 | |  |  |
| *Wilcoxon signed-rank test* | | ***P=0.0013*** | ***P=0.0041*** | | ***p=0.0005*** | |  |  |
| **PRE** | Number of events | 51 | 53 | | 295 | |  |  |
| **POST** | Number of events | 16 | 26 | | 217 | |  |  |
| Relative Reduction | | 69% | 51% | | 26% | |  |  |
| **Notes**:  **PRE**: year prior to commencing IDM  **POST:** year after IDM enrolment. Number of events is calculated from the rate ((rate*number of patients)/100), Rate is annualised ((events/ months of follow-up) *12*100)  **^1^**Visits to the emergency department that did not result in a hospital admission.  Significance level 0.05, p values adjusted for multiple testing using the Holm correction, bold indicates significance | | | | | | | |  |

**Supplemental Figure S3**: Acute health service use for heart failure, comparing the year prior to Best Care with annualised year post for **heart failure reduced ejection fraction** (**HFrEF)**.

| **168** individuals with at least 6 months of follow-up | | **Heart Failure Related:** | | | | | |  |
| --- | --- | --- | --- | --- | --- | --- | --- | --- |
|  |  | **Hospital Admissions** | | **Emergency Department Visits^1^** | | **Urgent Family Physician Visits** | | |
| Rate of events per 100 individuals with heart failure per year | | 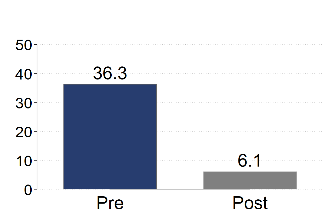 | 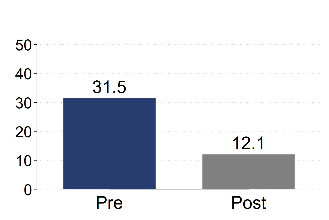 | | 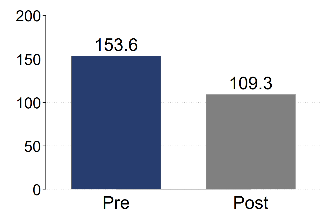 | |  |  |
| *Wilcoxon signed-rank test* | | ***p<0.0001*** | ***p=0.0004*** | | ***p=0.0036*** | |  |  |
| **PRE** | Number of events | 61 | 53 | | 258 | |  |  |
| **POST** | Number of events | 10 | 20 | | 184 | |  |  |
| Relative Reduction | | 84% | 62% | | 29% | |  |  |
| **Notes**:  Missing LVEF for 2/267 patients, unable to classify HFrEF/HFpEF  **PRE**: year prior to commencing IDM  **POST:** year after IDM enrolment. Number of events is calculated from the rate ((rate*number of patients)/100), Rate is annualised ((events/ months of follow-up) *12*100)  **^1^**Visits to the emergency department that did not result in a hospital admission.  Significance level 0.05, p values adjusted for multiple testing using the Holm correction, bold indicates significance | | | | | | | |  |

**Supplemental Figure S4**: Acute health service use for heart failure, comparing the year prior to Best Care with annualised year post for **heart failure preserved ejection fraction (HFpEF)**.

| **97** individuals with at least 6 months of follow-up | | **Heart Failure Related:** | | | | | |  |
| --- | --- | --- | --- | --- | --- | --- | --- | --- |
|  |  | **Hospital Admissions** | | **Emergency Department Visits^1^** | | **Urgent Family Physician Visits** | | |
| Rate of events per 100 individuals with heart failure per year | | 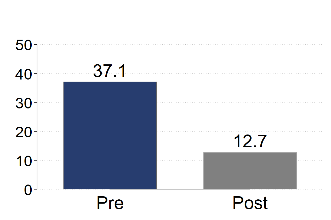 | 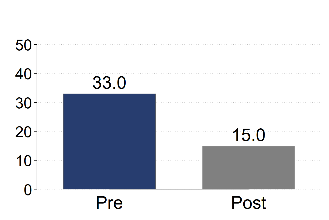 | | 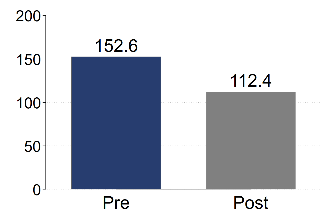 | |  |  |
| *Wilcoxon signed-rank test* | | ***p=0.0031*** | *p=0.0331* | | *p=0.0088* | |  |  |
| **PRE** | Number of events | 36 | 32 | | 148 | |  |  |
| **POST** | Number of events | 12 | 15 | | 109 | |  |  |
| Relative Reduction | | 67% | 53% | | 26% | |  |  |
| **Notes**:  Missing LVEF for 2/267 patients, unable to classify HFrEF/HFpEF  **PRE**: year prior to commencing IDM  **POST:** year after IDM enrolment. Number of events is calculated from the rate ((rate*number of patients)/100), Rate is annualised ((events/ months of follow-up) *12*100)  **^1^**Visits to the emergency department that did not result in a hospital admission.  Significance level 0.05, p values adjusted for multiple testing using the Holm correction, bold indicates significance | | | | | | | |  |

**Supplemental Figure S5.** Change in health-related quality of life (QoL), stratified by baseline score, in the **92** individuals with documented QoL scores at initial and at least one follow-up visit in community based **specialist care.
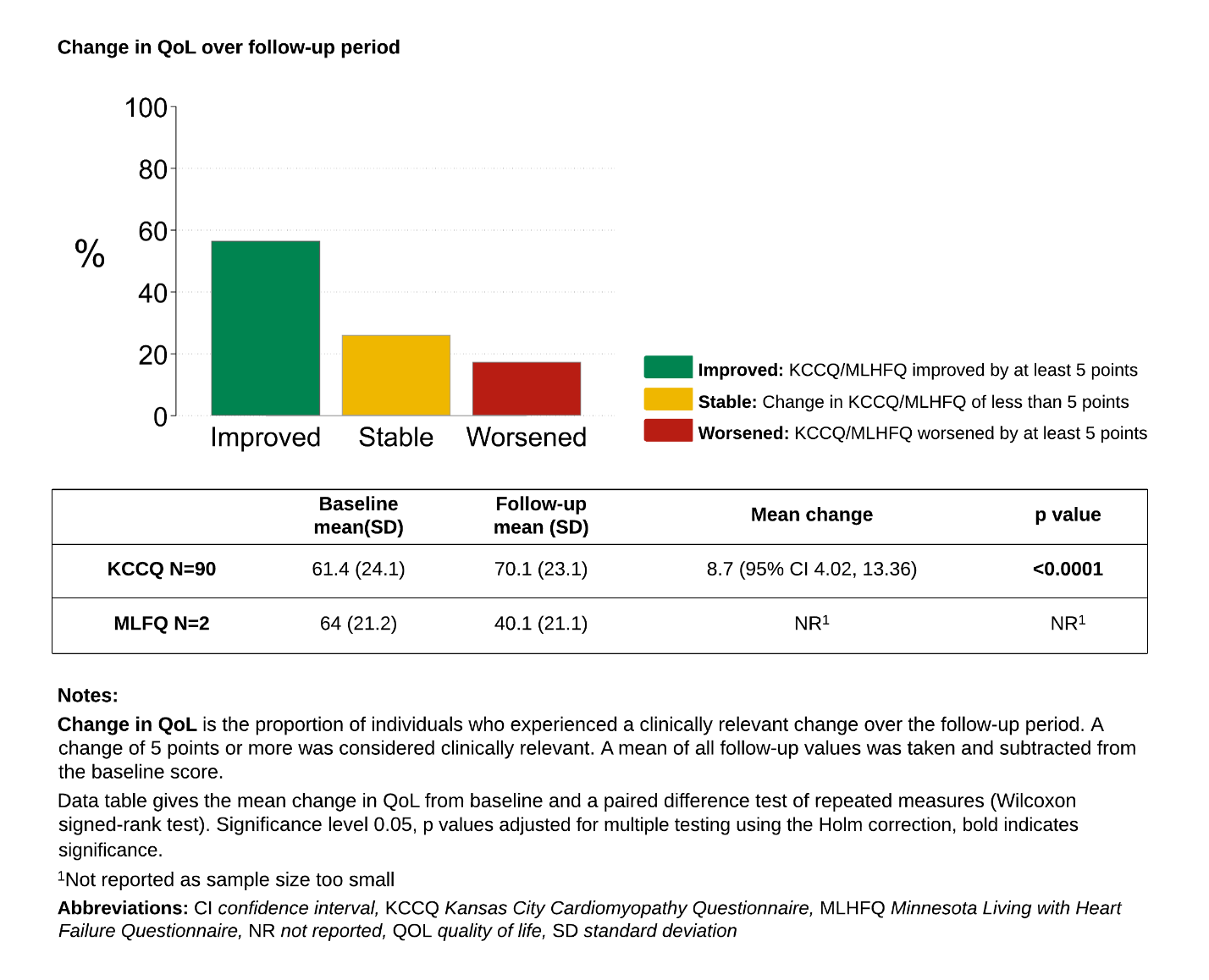
**

**Supplemental Figure S6.** Change in health-related quality of life (QoL), stratified by baseline score, in the **194** individuals with documented QoL scores at initial and at least one follow-up visit in **primary care**.

**
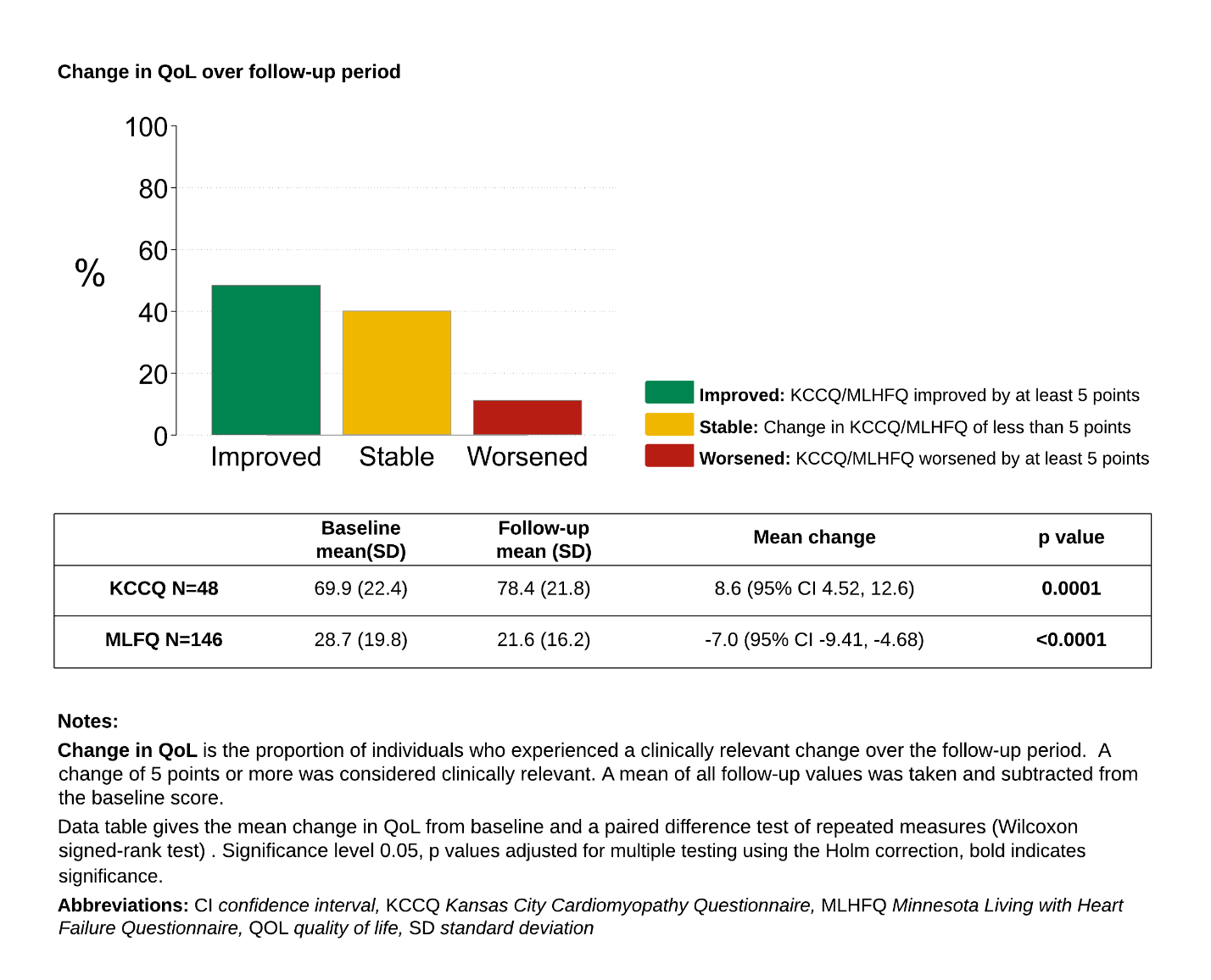
**

**Supplemental Figure S7.** Change in health-related quality of life (QoL), stratified by baseline score, in the **176** individuals with documented QoL scores at initial and at least one follow-up visit in **heart failure reduced ejection fraction (HFrEF).
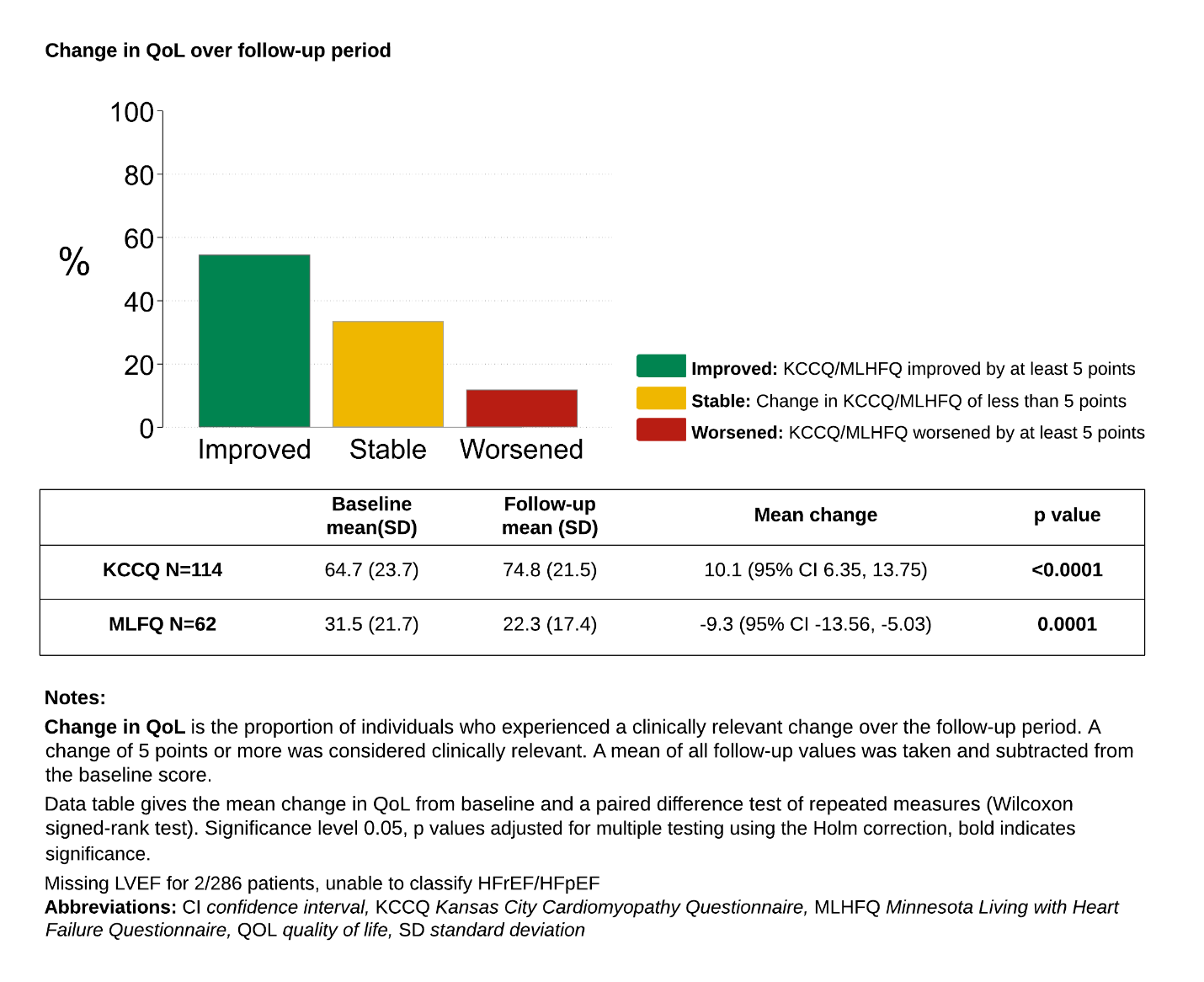
**

**Supplemental Figure S8.** Change in health-related quality of life (QoL), stratified by baseline score, in the **108** individuals with documented QoL scores at initial and at least one follow-up visit in **heart failure preserved ejection fraction (HFpEF)**.
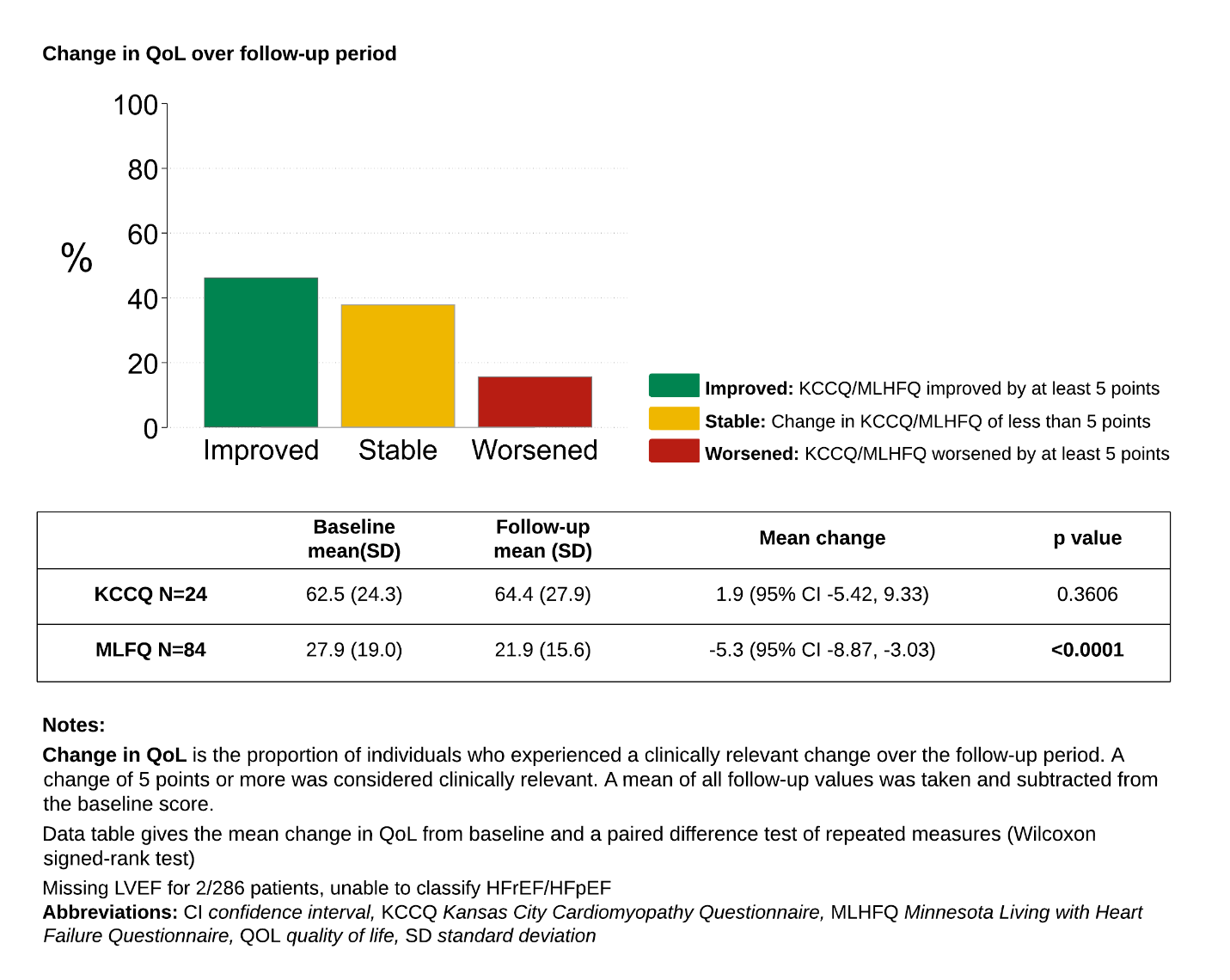


**Supplemental Table S1.** Sensitivity analysis comparing baseline characteristics of the complete cohort, patients excluded with <6months of follow-up, and patients included in the pre-post health service utilization outcome analysis.

| **Baseline Demographic and Clinical Characteristics** | | | |  | |
| --- | --- | --- | --- | --- | --- |
|  |  | **Complete Cohort**  **N=715** | **Excluded^5^**  **N=153** | | **HSU Pre-Post Analysis**  **N=267** |
| **Sex** | Male | 426 (59.6%) | 74 (48.4%) | | 164 (61.4%) |
|  | Female | 289 (40.4%) | 79 (51.6%) | | 103 (38.6%) |
| **Age (years)** | *Mean (SD)* | 73.5 (10.7) | 74.6 (11.3) | | 72.2 (11.5) |
| **Body mass index (kg/m^2^)** | *Mean (SD)* | 31.5 (7.7) | 31.0 (8.5) | | 31.9 (7.4) |
| **Racial Group** | Caucasian | 695 (97.2%) | 147 (96.1%) | | 258 (96.6%) |
| **Smoking Status** | Current smoker | 64 (9.0%) | 11 (7.1%) | | 27 (10.1%) |
| **Quality of Life** | **KCCQ (score 0-100)^1^** *Mean (SD)* | 66.6 (24.6) | 62.9 (29.0) | | 59.2 (23.7) |
|  | **MLHFQ (score 0-105)^2^** *Mean (SD)* | 29.1 (20.2) | 29.4 (19.5) | | 29.0 (20.6) |
|  | *Missing* | *84 (11.7%)* | *43 (28.1%)* | | *22 (8.2%)* |
| **Comorbidities** | 0 | 10 (1.4%) | 6 (3.9%) | | 1 (0.4%) |
|  | 1-2 | 125 (17.5%) | 37 (24.2%) | | 48 (18.0%) |
|  | >2 | 580 (81.1%) | 110 (71.9%) | | 218 (81.7%) |
| **Charlson Comorbidity Index^3^** | *Mean (SD)* | 5.4 (1.9) | 5.5 (1.6) | | 5.4 (2.2) |
|  | ≥5 | 328 (69.5%) | 43 (76.8%) | | 85 (70.2%) |
| **Seen by Specialist** | Cardiologist | 454 (63.5%) | 86 (56.2%) | | 175 (65.5%) |
|  | Internal Medicine | 124 (17.3%) | 26 (17.0%) | | 66 (24.7%) |
|  | None | 185 (25.9%) | 51 (33.3%) | | 54 (20.2%) |
| **IDM program** | Specialist-care clinic | 219 (30.6%) | 39 (25.5%) | | 107 (40.1%) |
|  | Primary-care clinic | 496 (69.4%) | 114 (74.5%) | | 160 (59.9%) |
| **HFrEF** | LVEF ≤45% | 429 (60.0%) | 66 (43.1%) | | 168 (63.4%) |
| **HFpEF** | LVEF >45% | 274 (38.3%) | 77 (50.3%) | | 97 (36.6%) |
|  | *Missing* | *12 (2.0%)* | *10 (6.5%)* | | *2 (0.8%)* |
| **Echocardiogram year prior** | | 616 (86.2%) | 211 (96.4%) | | 243 (91.0%%) |
| **NYHA** | I | 119 (16.6%) | 25 (16.3%) | | 34 (12.7%) |
|  | II | 356 (49.8%) | 73 (47.7%) | | 142 (53.18%) |
|  | III | 221 (30.9%) | 50 (32.7%) | | 87 (32.6%) |
|  | IV | 19 (2.7%) | 5 (3.3%) | | 4 (1.5%) |
| **Heart failure related health service use (year prior)** | |  |  | |  |
| **Hospital admissions** | Number of events | 263 | 71 | | 97 |
|  | Number of individuals | 202 (28.3%) | 54 (35.3%) | | 74 (27.7%) |
|  | Rate of events/100 patients/year | 36.8 | 46.4 | | 36.3 |
| **Emergency department visits** (not leading to admission) | Number of events | 214 | 62 | | 85 |
|  | Number of individuals | 154 (21.5%) | 40 (26.1%) | | 66 (24.7%) |
|  | Rate of events/100 patients/year | 29.9 | 40.5 | | 31.8 |
| **Urgent family physician visits** | Number of events | 924 | 212 | | 408 |
|  | Number of individuals | 323 (45.2%) | 63 (41.2%) | | 104 (39.0%) |
|  | Rate of events/100 patients/year | 129.2 | 138.6 | | 152.8 |
| **Medications** | ARNI (HFrEF only)^4^ | 186 (43.4%) | 26 (39.4%) | | 65 (38.7%) |
|  | ACEi/ARB | 293 (41.0%) | 48 (31.4%) | | 102 (38.2%) |
|  | Beta-blocker | 540 (75.5%) | 97 (63.4%) | | 199 (74.5%) |
|  | MRA | 254 (35.5%) | 37 (24.2%) | | 98 (36.7%) |
|  | SGLT2i | 168 (23.5%) | 4 (2.6%) | | 43 (16.1%) |
|  | Diuretic | 496 (69.4%) | 111 (72.6%) | | 196 (73.%) |
| **Notes:**  **^1^**KCCQ-23, scored 0-100 where 100 represents best quality of life. N=423, N=36 (excluded), N=111 (HSU pre-post analysis)  **^2^**MLHFQ, scored 0-105 where 105 represents the worst quality of life. N=208, N=74 (excluded), N=134 (HSU pre-post analysis)  **^3^**Charlson Co-morbidity Index self-reported since Sept 2020 (Age adjusted index reported), N=472, N=56 (excluded), N=21 (HSU pre-post analysis)  **^4^**Only HFrEF N=429, N=66 (excluded), N=168 (HSU pre-post analysis)  **^5^**153 patients were excluded due to mortality, discharge from specialist management, left the area, admitted to long-term care, stopped participation, and are still treated but on the Best Care COPD program, 267 were included in the analysis and 295 had not been enrolled on the program long enough to have acquired 6 months of follow-up data.  **Abbreviations:** ACEi *angiotensin-converting enzyme inhibitor,* ARB *angiotensin receptor blockers,* ARNI *angiotensin receptor/neprilysin inhibitor,* HFrEF *heart failure with reduced ejection fraction,* HFpHF *heart failure with preserved ejection fraction,* HSU *health service utilization,* KCCQ *Kansas City Cardiomyopathy Questionnaire,* LVEF *left ventricular ejection fraction,* MLHFQ *Minnesota Living with Heart Failure Questionnaire,* MRA *mineralocorticoid receptor antagonist,* SD *standard deviation,* SGLT2i *sodium-glucose cotransporter-2 inhibitor,* NYHA *New York Heart Association* | | | | | |

**Supplemental Table S2.** Sensitivity analysis comparing baseline characteristics of the complete cohort, the patients excluded with missing quality of life (QoL) scores, and the patients included in the pre-post QoL outcome analysis.

| **Baseline Demographic and Clinical Characteristics** | | | |  | |
| --- | --- | --- | --- | --- | --- |
|  |  | **Complete Cohort**  **N=715** | **Missing QoL Scores^5^**  **N=232** | | **Qol Pre-Post Analysis**  **N=286** |
| **Sex** | Male | 426 (59.6%) | 140 (60.3%) | | 175 (61.2%) |
|  | Female | 289 (40.4%) | 92 (39.7%) | | 111 (38.8%) |
| **Age (years)** | *Mean (SD)* | 73.5 (10.7) | 73.8 (11.2) | | 72.5 (10.5) |
| **Body mass index (kg/m^2^)** | *Mean (SD)* | 31.5 (7.7) | 30.8 (7.5) | | 32.0 (7.9) |
| **Racial Group** | Caucasian | 695 (97.2%) | 225 (97.0%) | | 279 (97.6%) |
| **Smoking Status** | Current smoker | 64 (9.0%) | 27 (11.6%) | | 21 (7.3%) |
| **Quality of Life** | **KCCQ (score 0-100)^1^** *Mean (SD)* | 66.6 (24.6) | NA | | 64.3 (23.8) |
|  | **MLHFQ (score 0-105)^2^** *Mean (SD)* | 29.1 (20.2) | NA | | 29.1 (20.2) |
|  | *Missing* | *84 (11.7%)* | NA | | - |
| **Comorbidities** | 0 | 10 (1.4%) | 4 (1.7%) | | 2 (0.7%) |
|  | 1-2 | 125 (17.5%) | 42 (18.1%) | | 51 (17.8%) |
|  | >2 | 580 (81.1%) | 186 (80.2%) | | 233 (81.5%) |
| **Charlson Comorbidity Index^3^** | *Mean (SD)* | 5.4 (1.9) | 5.6 (1.9) | | 5.2 (2.0) |
|  | ≥5 | 328 (69.5%) | 128 (73.6%) | | 94 (68.1%) |
| **Seen by Specialist** | Cardiologist | 454 (63.5%) | 139 (59.9%) | | 189 (66.1%) |
|  | Internal Medicine | 124 (17.3%) | 28 (12.1%) | | 65 (22.7%) |
|  | None | 185 (25.9%) | 78 (33.6%) | | 59 (20.6%) |
| **IDM program** | Specialist care clinic | 219 (30.6%) | 70 (30.2%) | | 92 (32.2%) |
|  | Primary care clinic | 496 (69.4%) | 162 (69.8%) | | 194 (67.8%) |
| **HFrEF** | LVEF ≤45% | 429 (60.0%) | 137 (59.1%) | | 176 (61.5%) |
| **HFpEF** | LVEF >45% | 274 (38.3%) | 88 (37.9%) | | 108 (37.8%) |
|  | *Missing* | *12 (2.0%)* | *7 (3.0%)* | | *2 (0.7%)* |
| **Echocardiogram year prior** | | 616 (86.2%) | 211 (96.4%) | | 259 (90.6%) |
| **NYHA** | I | 119 (16.6%) | 35 (15.1%) | | 40 (14.0%) |
|  | II | 356 (49.8%) | 115 (49.6%) | | 145 (50.7%) |
|  | III | 221 (30.9%) | 76 (32.8%) | | 95 (33.2%) |
|  | IV | 19 (2.7%) | 6 (2.6%) | | 6 (2.1%) |
| **Heart failure related health service use (year prior)** | |  |  | |  |
| **Hospital admissions** | Number of events | 263 | 93 | | 103 |
|  | Number of individuals | 202 (28.3%) | 70 (30.2%) | | 79 (27.6%) |
|  | Rate of events/100 patients/year | 36.8 | 40.1 | | 36.0 |
| **Emergency department visits** (not leading to admission) | Number of events | 214 | 69 | | 92 |
|  | Number of individuals | 154 (21.5%) | 42 (18.1%) | | 74 (25.9%) |
|  | Rate of events/100 patients/year | 29.9 | 29.7 | | 32.2 |
| **Urgent family physician visits** | Number of events | 924 | 285 | | 415 |
|  | Number of individuals | 323 (45.2%) | 126 (54.3%) | | 113 (39.5%) |
|  | Rate of events/100 patients/year | 129.2 | 122.8 | | 145.1 |
| **Medications** | ARNI (HFrEF only)^4^ | 186 (43.4%) | 61 (44.5%) | | 62 (35.2%) |
|  | ACEi/ARB | 293 (41.0%) | 89 (38.4%) | | 121 (42.3%) |
|  | Beta-blocker | 540 (75.5%) | 166 (71.6%) | | 214 (74.8%) |
|  | MRA | 254 (35.5%) | 75 (32.3%) | | 107 (37.4%) |
|  | SGLT2i | 168 (23.5%) | 51 (22.0%) | | 58 (20.3%) |
|  | Diuretic | 496 (69.4%) | 160 (69.0%) | | 206 (72.0%) |
| **Notes:**  **^1^**KCCQ-23, scored 0-100 where 100 represents best quality of life.  **^2^**MLHFQ, scored 0-105 where 105 represents the worst quality of life.  **^3^**Charlson Co-morbidity Index self-reported since Sept 2020 (Age adjusted index reported), N=472, N=174 (missing QoL), N=138 (QoL pre-post analysis)  **^4^**Only HFrEF N=429, N=137 (missing QoL), N=176 (QoL pre-post analysis)  **^5^**232 had missing QoL scores, 286 were included in the analysis, and 197 did not have any missing data but only had an initial visit.  **Abbreviations:** ACEi *angiotensin-converting enzyme inhibitor,* ARB *angiotensin receptor blockers,* ARNI *angiotensin receptor/neprilysin inhibitor,* HFrEF *heart failure with reduced ejection fraction,* HFpHF *heart failure with preserved ejection fraction,* KCCQ *Kansas City Cardiomyopathy Questionnaire,* LVEF *left ventricular ejection fraction,* MLHFQ *Minnesota Living with Heart Failure Questionnaire,* MRA *mineralocorticoid receptor antagonist,* SD *standard deviation,* SGLT2i *sodium-glucose cotransporter-2 inhibitor,* NYHA *New York Heart Association* | | | | | |
